# Three-compartment (3C) pharmacokinetic modeling is more accurate than two-compartment (2C) modeling of myocardial fibrosis gadolinium kinetics

**DOI:** 10.1186/1532-429X-14-S1-P248

**Published:** 2012-02-01

**Authors:** James W Goldfarb, Wenguo Zhao, Jing Han

**Affiliations:** 1 Research and Education, St Francis Hospital, Roslyn, NY, USA; 2 Biomedical Engineering, SUNY Stony Brook, Stony Brook, NY, USA

## Summary

We observed significantly different curve shapes in healed chronic myocardial infarction when compared with normal myocardium and found that gadolinium kinetics was more accurately modeled (greater R2) with a 3C model rather than a 2C model. 2C Modeling of fibrotic and normal myocardium showed both a significant difference between the transport into the tissue and extracellular volumes while 3C modeling showed only a significant difference between the extracellular volumes of fibrotic and normal myocardium as well as the functional existence of a third compartment in fibrotic and not in normal myocardium.

## Background

There is increased interest in the quantitative assessment of myocardial gadolinium enhancement. There are a number of preclinical and clinical studies that have show the utility of MR derived variables for the assessment of dense as well as diffuse myocardial fibrosis. These studies use blood and myocardial T1 measurements followed by calculations of blood-tissue partition coefficients and tissue fractional volumes (gadolinium volume of distribution (Vd) and extracellular volume (Ve)). The typical assumption is that two-compartmental myocardial tissue modeling is sufficient. This assumes that the gadolinium based contrast agent (GBCA) freely passes into and out of and freely distributes within both normal and diseased myocardial tissue. Most publications assume a 2C model where only the volume of distribution changes in fibrotic myocardium. With this assumption, the GBCA time course curve shapes should remain the same and only the GBCA concentration and hence image signal should increase as there is increased GBCA per unit tissue of myocardium.

## Methods

**Figure 1:**
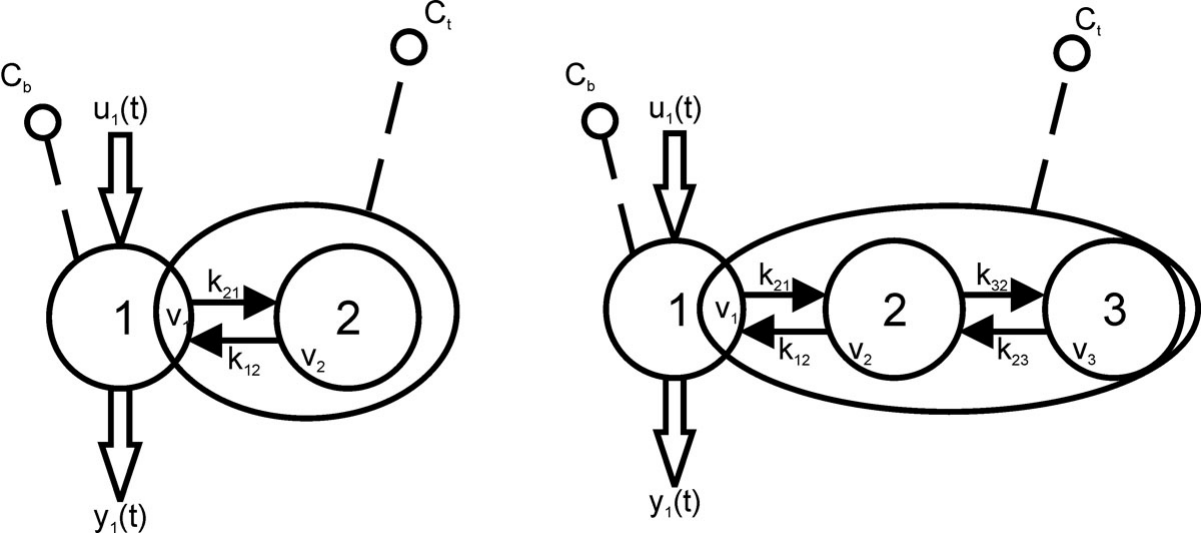
Two- and three-compartment models. Compartment 1: Blood; Compartment 2: Extracellular space with communication with blood (free compartment); Compartment 3: Extracellular Space without communication with blood (trapping compartment). Note that compartment three may not be a physical compartment. Inputs to the model are the blood Gd concentration (Cb) and the myocardial tissue Gd-concentration (Ct). Calculated model parameters are the transfer constant between compartments (k) and fractional volumes (v).

Twenty-five individuals (23 men and two women; age mean±std, 61.5±9.9 years) underwent MR imaging at 1.5T. All subjects in this study had a prior SPECT study as part of their routine medical care and the diagnosis of myocardial infarction. The infarct age ascertained from medical history was on average 11.6±10.1 years and ranged from 2 to 31 years. Single slice T1 measurements were performed before contrast administration and after injection of 0.2 mmol/kg of gadodiamide, approximately every two minutes using an inversion recovery CINE balanced steady-state free precession technique. T1 values of blood, normal and fibrotic myocardium were calculated and converted to GBCA concentration. These values were fitted with 2C and 3C models (Fig 1). The model parameters and goodness of fit (R2) were compared with a Student’s t-test between fibrotic and normal myocardium as well as 2C and 3C models.

## Results

**Figure 2:**
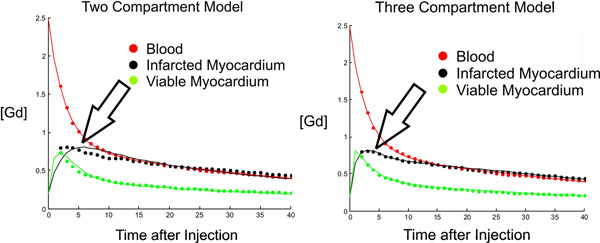
Gd concentration curves showed significantly different shapes of infarcted and viable myocardium. There was a significantly better fit for infarcted myocardial tissue with the three-compartment model (B) when compared to the two-compartment model (A). Viable myocardium was equally well fit with both models.

There was a clear difference in [GBCA] curves of healed infarction and viable myocardium (Fig 2A). There was a significantly better fit for infarcted tissue, (R2(3C) = .99 vs. R2(2C) = .77, p=0.001) with the 3C model when compared to the 2C model (Fig 2B). With 2C modeling, myocardial infarction was seen as a change in transport in to the tissue (k21, p=0.004) and extracellular volume (v2, p<0.0001). With 3C modeling, myocardial infarction was seen only as a difference in the transport of the GBCA into a functional third compartment (k32>0, p=0.0012) and not a difference in transport of GBCA into the tissue (k21, p=0.9452).

## Conclusions

3C modeling of GCBA kinetics provides a more accurate fit to modeling of healed myocardial infarction. There may not be a physical third compartment, but the better fit could suggest trapping of GBCA in fibrotic myocardium, possibly due to GBCA binding.

## Funding

American Heart Association Scientist Development Grant, 0635029N.

